# Impact of a pharmacist-led patient-centred care intervention along with textmessage reminders, on the management of newly diagnosed tubercular patients: A protocol for a randomized controlled trial

**DOI:** 10.12669/pjms.40.3.5356

**Published:** 2024

**Authors:** Farman Ullah Khan, Faiz Ullah Khan, Khezar Hayat, Yu Fang

**Affiliations:** 1 Farman Ullah Khan, Department of Pharmacy Administration and Clinical Pharmacy, Xi’an Jiaotong University, Xi’an, China; 2 Faiz Ullah Khan, Department of Pharmacy Administration and Clinical Pharmacy, Xi’an Jiaotong University, Xi’an, China; 3 Khezar Hayat, Department of Pharmacy Administration and Clinical Pharmacy, Xi’an Jiaotong University, Xi’an, China; 4 Yu Fang, Department of Pharmacy Administration and Clinical Pharmacy, Xi’an Jiaotong University, Xi’an, China

**Keywords:** Randomized controlled trial, Pharmacist intervention, Tuberculosis, Medication adherence

## Abstract

**Objectives:**

Non-adherence to tuberculosis (TB) treatment is the leading cause of the increase in drug resistance cases. This study will determine the effectiveness of pharmaceutical-care-based interventions coupled with short messages delivered by a pharmacist on treatment outcomes and adherence among TB patients.

**Methods:**

The study will be conducted in TB Control Center of Pakistan Institute of Medical Sciences Hospital, Islamabad and District Bannu TB Control Center time period will be from August 2019 to September 2021. The patients will be included into the control group (usual care) or the intervention group pharmaceutical care and SMS reminder. The primary outcome includes a change in mean score from baseline in treatment outcomes and adherence, measured by Morisky Medication Adherence Scale, and clinic appointment attendance registration. Secondary outcomes include health-related quality of life of patients, disease knowledge, and patient satisfaction with the intervention.

**Result:**

The major issues in patients with TB are cure rate and medication adherence. The method anticipated in this manuscript could set the foundation of pharmaceutical care and mobile SMS for the future provision of care to improve TB treatment outcomes.

**Conclusion:**

The study will make available fundamental information about the influence of the patient centered program on the adherence and clinical outcomes of patients with TB.

***Trial status and registration:*** Clinical Trials.gov assigned Identifier NCT04645836.

## INTRODUCTION

Tuberculosis (TB) is a leading public health issue globally, and nearly ten million people suffered from TB in 2019, as reported by the World Health Organization (WHO). All over the world, 30 high burden countries are responsible for 87% of all new TB cases each year. Pakistan was ranked fifth in 2019 due to the considerable number (562000 cases) of TB with 15000 cases of drug-resistant TB each year.^1^ This increase in the emergence of drug-resistance TB cases is possibly due to nonadherence, poor follow-up, lack of social and motivational support during the treatment.^2^ Non-adherence to TB treatment remains an essential factor for increasing drug-resistant TB cases in developing countries, including Pakistan.^3^ According to Akhtar et al., drug-resistant TB case is more prevalent in Pakistan in the reproductive age group (57%) and in urban areas (97%).^4^ Directly observed treatment support DOTS strategy has been established to resolve all problems that could arise during treatment. Still, this strategy has numerous barriers to optimal implementation, especially in developing countries, including poor economic conditions, large population density, long travel distances, fragile healthcare system, and shortage of human resources.^3^

Available literature suggests that there has been a constant increase in electronic gadgets throughout the world, including developing countries. Along with mobile equipment technologies, another pharmaceutical care-led strategy is the provision of instructions to patients on illness, medication use, and medication-related issues by a pharmacist.^5^-^7^ Pharmacists play a fundamental part in the management of patients chronic disease through pharmaceutical care. During the administration of pharmaceutical care treatment, the responsible pharmacist provides a cell phone number to patients and encourages them to call if they need any disease management advice.^8^ Recently, the most effective means of communication between patients and healthcare providers have been mobile phone messages, which are very economical and useful in both urban and rural areas.^9^,^10^ Therefore, in Pakistan, the TB control programme immediately needs a suitable and affordable course of action to improve treatment adherence. The current study was designed to determine the effectiveness of a pharmaceutical-care-based intervention coupled with short messages delivered by a pharmacist on treatment outcomes and adherence among TB patients.

## METHODS

### Trial Design:

**Fig.1:**
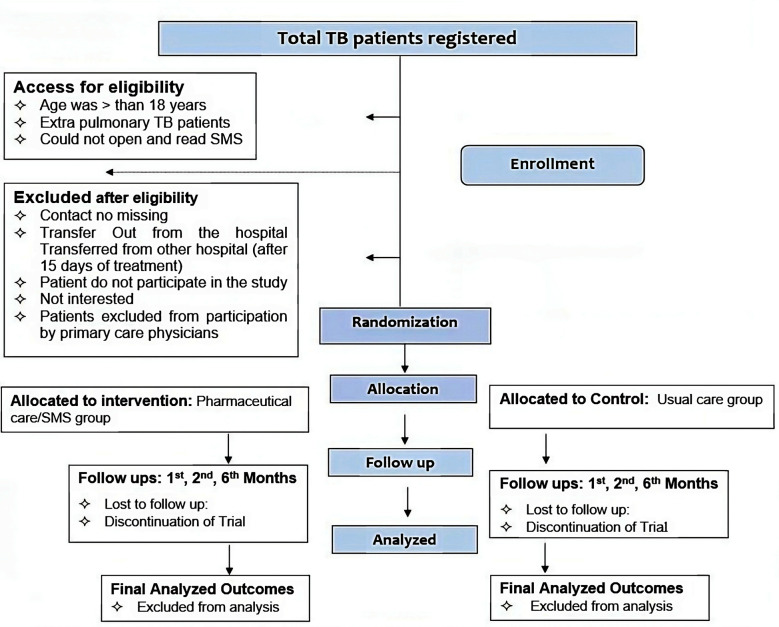
Study flow diagram.

This trial is a single-blind, randomized parallel study among all eligible new TB patients who are registered and receiving their treatment from the TB Control Center of the Pakistan Institute of Medical Sciences Hospital (PIMS) and District Bannu TB control program (DBTC). The time period will be from August 2019 to September 2021. The study will be randomized into two parallel groups (ratio 1:1): an intervention group (in which patients will be given pharmaceutical care and SMS reminders) versus a control group or a usual care group where participants will receive the same routine standard treatment care as the intervention group, but no pharmaceutical care and SMS text messages will be made (Fig.1).^11^

### Primary Objective:

The primary objective of this study is to assess the effectiveness of pharmaceutical care coupled with short messages provided by the pharmacist on treatment outcomes and adherence in newly diagnosed TB patients.

### Secondary Objectives:

Determine whether the patient’s quality of life,score increases in intervention arm patients from baseline to successful completion of treatment. In the intervention arm patient satisfaction feedback response will be assessed.

### Treatment Outcomes:

According to WHO guidelines^12^, treatment outcomes are classified as successful when the TB patient finalizes the treatment medication with an indication of cure and complete. The unsuccessful treatment outcomes are categorized into treatment failure, treatment defaulters, transfer out, or those registered patients whose outcomes are not known after completing the course.

### Level of Adherence:

The 8-item Morisky Medication Adherence Scale (MMAS-8) scale will be used for the measure of adherence rate. The initial seven questions of the MMAS-8 adherence scale have dichotomous (yes/no) answers, while the following five question offer a Likert reaction.^13^ Our study analysis team agreed that a patient will be considered adherent at the end of the trial if they have an MMAS-8 ≥6 or “non-adherent” if the MMAS-8 is less then 6 and also patient follow ups clinic appointment attendance will be checked. ***To measure HRQoL*** (Health-related Quality of Life), Re validated data assortment tool, European Quality of Life Scale EQ 5D 3L.^14^ ***Satisfaction*** with information can be assessed with the patient satisfaction feedback response questionnaire Table-I.

**Table-I:** Primary and secondary outcomes covered in the trial.

Primary outcomes	Measurement tool
Treatment Outcomes	Successful Outcomes, Cure, and Completed Treatment Unsuccessful Outcomes, Failure, Loss to follow-up, Deaths Unevaluated
Adherence	Morisky Medication Adherence Scale
Secondary outcomes	
Health-Related Quality of Life	European Quality of Life Scale EQ 5D 3L
Patient satisfaction	Patient Satisfaction Feedback Response questionnaire.

### Inclusion criteria:

- Men and women with age >= 18 years,
- Newly bacteriological confirmed TB cases (less than a month since diagnosis). This restriction (not more than one-month treatment) does not refer to patients whose most recent treatment outcome was a failure and assigned to a new treatment regimen.
- Have a mobile phone and can receive SMS messages or phone calls
- An address or residence location is readily accessible for visiting and willingness to inform the study team of any change of address during the treatment and follow-up period for at least 9 months.
- Willingness to comply with study procedures and provide written informed consent before study enrollment.

### Exclusion criteria:

- Who are critically ill,
- Have a physical disability that prevents full participation in the study such as vision, hearing, physically challenged, unable to answer questions
- Pregnant females,
- Patients receiving treatment from private clinics (who are not registered in the government TB sectors, and they seek care from private health care facilities).

### Intervention Group:

The study will be conducted among all eligible new TB patients who are registered and receiving their treatment from the TB Control Center of PIMS and DBTC. In the intervention group, the participant’s pharmacist will provide pharmaceutical care education at each follow-up visit and SMS reminders will be sent to patients in local and in national language. The messages will be aimed ‘to take their prescribed anti-TB drugs daily and record the behaviour change during the therapy.

### Phases of pharmacist counselling:

After documentation of base-line data, patient care interventions will be implemented. Pharmaceutical care will be provided face to face during monthly follow ups visits to health care center. Interventions will be divided into non-pharmacological and pharmacological. Non-pharmacological interventions included guidance on the proper use of drugs and their risks and pharmacological interventions included suspension, addition, or change in medication. If pharmacological interventions seem to be necessary, a collaborative decision will be made by the pharmacist, physician, and patient. The pharmacist will talk over the patient-related barriers to adherence, incomplete information related to medication and disease, and other drug-related problems.The pharmaceutical care and face-to-face counselling to the TB patients will be provided during the first interview, within two weeks of initiating of anti-tuberculosis treatment. The second interview will be conducted within two months. The third interview will be at the six month of the treatment and the final data will be collected before the final sputum test.

### Blinding:

Due to this study’s nature, it will not be possible that the patients and will remain entirely blinded to the principal investigator because they will receive SMS reminders and pharmaceutical care. Data collector and analysis team or other study-related pharmacists carrying out study outcome measurements such as treatment outcomes, adherence, quality of life and satisfaction questionnaire, will remain fully blinded to the allocation of the control and intervention group Table-II.

**Table-II:** Overall binding scheme

Stakeholders	Allocation scheme	Pharmacist Intervention	SMS Sender	Outcome assessment	Data analysis
Trial observer	Aware	Aware	Aware	Aware	Aware
Participants	Aware	Aware	Aware	Not aware	Not aware
Pharmacists	Aware	Aware	Aware	Not aware	Not aware
Pulmonologists	Aware	Aware	Aware	Not aware	Not aware
Data collectors	Not aware	Not aware	Not aware	Not aware	Not aware
Data analysis	Not aware	Not aware	Not aware	Not aware	Aware

### Sample size:

Considering the drop out in the study up to 10%, the intended sample size is 413 subjects per group is needed. Over a six month period between the groups, with a two-tailed α of 0.05 and (1-β) of 0.80 using the formula the total intended sample size is 426 patients for both groups.

### No intervention group:

The control group patient will be treated according to national TB control program recommendations. Control group participants will receive the same routine standard treatment care as the intervention group, but no pharmaceutical care and SMS text messages will be made. In Pakistan tuberculosis treatment usually includes daily self-management of medicine so no pharmacists are engaged in TB control centers. Therefore we have designed this trial to include a pharmacist in the circle of care of TB to decrease the rate of resistance of relapse cases and improve treatment outcomes.

### Statistical analysis and ethics approval:

The association between intervention and control groups the cure rates will be analyzed through a logistic regression model (odds ratio and 95% CI). Mann-Whitney U test will be used for continuous variables and chi-square for categorical variables. The study was subjected to two external peer reviews by the committee of the Pakistan Institute of Medical Sciences Hospital, Islamabad and Shaheed Zulfiqar Ali Medical University, Islamabad (F.1-1/2015/ERB/SZABMU/359) and ethically approved after scientific review. The study was also approved by Xian Jiaotong University, Health Science Center Biology Scientific and Research Ethics Committee (2019-1257).

## DISCUSSION

The major issues in patients of TB are cure rate and medication adherence. Therefore, this randomized trial, the first of its kind in Pakistan, aimed to investigate the impact of pharmacist-led pharmaceutical care in combination with a personalized SMS reminder on the rate of cure among TB patients along with their medication adherence. Because the rate of adherence and cure for tuberculosis infection remains questioned among TB patients.

Nowadays, in every disease, the demand for home-based management and self-care of the disease is increasing. However, if the patients fail to understand the management of the disease by themselves, then it may lead to non-adherence and mobility problems, which will increase the risk of complications. Hence, the quality of life and cure rate will be reduced.^8^,^15^ Therefore, in the current situation, it is essential that patient-centred care approaches must be applied to address and manage the problems arising from chronic diseases.^16^ Furthermore, two RCTs in a chronic condition such as hypertension were conducted in Pakistan, which showed that PC intervention has significantly enhanced treatment outcomes, disease knowledge, medication adherence, and HRQoL.^11^ Pharmacists are extremely qualified healthcare experts due to the importance of patient-centred care^17^,^18^ Pharmacists are at perfect position to provide certain mobile health services, with medication intake as a part of their daily practice.^19^,^20^

There are several limitations to this RCT. First, we will use patient self-reports to measure medication adherence, which could be overestimated. However, the reliability and validity of the MMAS-8 are high, and this scale is widely used across the world to measure medication adherence. Second, there is the probability of selection bias of TB participants, since in the study only those participants are enrolled, who have a mobile phone and also they are willing to text. Communication between a pharmacist and patients is also very important. If a pharmacist has good interpersonal and communication skills and can build trust with the patients, and they will have better treatment adherence.

### Limitations:

This limitation is understood, but because the main objective of this study is to conceptualize the role of the pharmacist in TB care, therefore to overcome this limitation, the pharmacist will remain completely blind to primary and secondary study outcomes.

## CONCLUSION

The study will make available fundamental information about the influence of the patient centered program on the adherence and clinical outcomes of patients with TB.

## References

[1] WHO (2020). Glob. Tuberc. Rep.

[2] WHO EMRO Tuberculosis 2018. |Tuberculosis |Programmes |Pakistan [Internet].

[3] Khan FU, Asghar Z, Tipu MK, Asim Ur Rehman, Khan A, Tofeeq Ur-Rehman (2021). Effect of displacement on Adherence to TB Treatment:An observational study in TB patients from Internally Displaced Persons of Pakistan. Pak J Med Sci.

[4] Akhtar AM, Arif MA, Kanwal S, Majeed S (2016). Prevalence and drug resistance pattern of MDR TB in retreatment cases of Punjab, Pakistan. J Pak Med Assoc.

[5] Horvath T, Azman H, Kennedy GE, Rutherford GW (2012). Mobile phone text messaging for promoting adherence to antiretroviral therapy in patients with HIV infection. Cochrane Database Syst Rev.

[6] Thirumurthy H, Lester RT (2012). M-health for health behaviour change in resource-limited settings:applications to HIV care and beyond. Bulletin World Health Organization.

[7] Clark PM, Karagoz T, Apikoglu-Rabus S, Izzettin FV (2007). Effect of pharmacist-led patient education on adherence to tuberculosis treatment. Am J Health Syst Pharm.

[8] Kayhan S, Akguuml A (2011). Therapeutic monitoring of isoniazid, rifampicin, ethambutol and pyrazinamide serum levels in the treatment of active pulmonary tuberculosis and determinants of their serum concentrations. Afr J Pharmacy Pharmacol.

[9] Hall CS, Fottrell E, Wilkinson S, Byass P (2014). Assessing the impact of mHealth interventions in low-and middle-income countries-what has been shown to work?. Global Health Action.

[10] Schulz KF, Altman DG, Moher D CONSORT 2010 statement:Updated guidelines for reporting parallel group randomized trials. Ann Intern Med.

[11] WHO (2016). World Health Oraganziation treatment guidelines for drug-resistant tuberculosis 2016 updated. Accessed on 20 october 2020 Available on www.apps.who.int. World Health Organization.

[12] Morisky DE, Ang A, Krousel-Wood M, Ward HJ (2008). Predictive validity of a medication adherence measure in an outpatient setting. J Clin Hypertens.

[13] Herdman M, Gudex C, Lloyd A, Janssen M, Kind P, Parkin D Development and preliminary testing of the new five-level version of EQ-5D (EQ-5D-5L). Qual Life Res.

[14] Sadaf R, Munir T, Farrukh S, Abbasi S (2020). Prevalence of latent tuberculosis infection in healthcare workers in tertiary care hospitals of Pakistan. Pak J Med Sci.

[15] Bukhsh A, Nawaz MS, Ahmed HS, Khan TM (2018). A randomized controlled study to evaluate the effect of pharmacist-led educational intervention on glycemic control, self-care activities and disease knowledge among type 2 diabetes patients:A consort compliant study protocol. Medicine.

[16] Shah A, Naqvi AA, Ahmad R (2016). The need for providing pharmaceutical care in geriatrics:A case study of diagnostic errors leading to medication-related problems in a patient treatment plan. Arch Pharm Pract.

[17] Chertes A, Crisan O (2019). Standards for good pharmacy practice-a comparative analysis. Farm Clin.

[18] Ivey MF (2019). Global opportunity:Pharmacists working together to improve patient care. Am J Health Syst Pharm.

[19] Swieczkowski D, Merks P, Gruchala M, Jaguszewski MJ (2016). The role of the pharmacist in the care of patients with cardiovascular diseases. Kardiol Pol.

[20] Haramiova Z, Stasko M, Hulin M, Tesar T, Kuzelova M, Morisky DM (2017). The effectiveness of daily SMS reminders in pharmaceutical care of older adults on improving patients'adherence to antihypertensive medication (SPPA):study protocol for a randomized controlled trial. Trials.

